# The Adjuvant Effects on Vaccine and the Immunomodulatory Mechanisms of Polysaccharides From Traditional Chinese Medicine

**DOI:** 10.3389/fmolb.2021.655570

**Published:** 2021-04-01

**Authors:** Danyang Wang, Yonghui Liu, Wei Zhao

**Affiliations:** State Key Laboratory of Medicinal Chemical Biology, Key Laboratory of Molecular Drug Research and KLMDASR of Tianjin, College of Pharmacy, Nankai University, Tianjin, China

**Keywords:** polysaccharides, vaccine, adjuvant, immunomodulatory, signaling pathway, TCM

## Abstract

Vaccination is still the most successful strategy to prevent and control the spread of infectious diseases by generating an adequate protective immune response. However, vaccines composed of antigens alone can only stimulate weak immunogenicity to prevent infection in many cases. Adjuvant can enhance the immunogenicity of the antigens. Therefore, adjuvant is urgently needed to strengthen the immune response of the vaccines. An ideal adjuvant should be safe, cheap, biodegradable and biologically inert. In addition to having a long shelf life, it can also promote cellular and humoral immune responses. Traditional Chinese medicine (TCM) has many different ingredients, such as glycosides, polysaccharides, acids, terpenes, polyphenols, flavonoids, alkaloids, and so on. TCM polysaccharides are one of the main types of biologically active substances. They have a large range of pharmacological activities, especially immunomodulatory. TCM polysaccharides can regulate the immune system of animals by binding to multiple receptors on the surface of immune cells and activating different signal pathways. This review focuses on a comprehensive summary of the most recent developments in vaccine adjuvant effects of polysaccharides from many important TCM, such as *Artemisia rupestris L., Cistanche deserticola, Pinus massoniana, Chuanminshen violaceum, Astragalus*, *Ganoderma lucidum, Codonopsis pilosula, Lycium barbarum, Angelica, Epimedium, and Achyranthes bidentata.* Moreover, this review also introduces their immunomodulatory effects and the molecular mechanisms of action on animal bodies, which showed that TCM polysaccharides can activate macrophages, the signal pathway of T/B lymphocytes, regulate the signal pathway of natural killer cells, activate the complement system, and so on.

## Introduction

Vaccination, as the most effective intervention in modern medicine, can greatly reduce mortality and extend life expectancy (Pourseif et al., 2021). It has changed the lives of millions of people and animals. Vaccines based on inactivated pathogens, attenuated live pathogens, surface molecules such as carbohydrates, proteins, and lipids, or recombinant antigens can induce neutralizing antibodies against particular pathogens through subcutaneous, intramuscular, oral or intranasal administration (Lamas et al., 2008).

Vaccines composed of antigens only are effective in some cases, but in many cases, they can only stimulate weak immunogenicity to prevent infection and cannot induce sufficient protective immune responses. Therefore, vaccine adjuvants are needed to enhance the immune response in the preparation of vaccines (Korsholm, 2011). Proper adjuvants should include the following characteristic properties, such as, making vaccine more cost effective (fewer doses required), effective innate immune signals (including danger signals), good immunomodulatory capacity, long-lasting adaptive immune response, generation of cytotoxic T cells, antigen-specific clonal expansion, high specific antibody production, and making antigen more potent (less dose required) (Reed et al., 2020).

Polysaccharides from TCM are a recent research hotspot. They are biological macromolecules composed of 10 or more monosaccharides, with different structures and sugar components (Xie et al., 2015). TCM polysaccharides are a group of natural substances, there are many advantages, such as high safety, high drug resistance, increased humoral, and cellular immunity, non-toxic side effects, low side effects, promote humoral immunity, exact effect, wide range of medication (Leung et al., 2006). Recent studies have shown that TCM polysaccharides have a variety of biological effects. For example, *Acanthopanax* polysaccharides and *Astragalus* polysaccharide have immunomodulatory, antiviral, anti-tumor, antioxidant, antimutagenic, anti-diabetic, antibiotic anticoagulant, and anti-inflammatory activities (Xie et al., 2015, 2016b; Yang et al., 2020). The regulation of immune cells by TCM polysaccharides is closely related to the regulation of cell signal transduction functions. By mediating cell signal pathways and then regulating gene expression in cells, the regulation of apparent traits was achieved (Jiang et al., 2010).

A large number of bioactive polysaccharides from different medicinal plant sources of physical and chemical properties constitute a large number of material sources for future applications, especially medicinal applications. They have attracted widespread attention in vaccine preparations and have become the development trend of vaccine adjuvants. These characteristics of polysaccharides have drawn attention of many scientists to explore the potential to develop polysaccharides as successful vaccine adjuvants.

This review summarizes the recent research on TCM polysaccharides as adjuvants for human or animal vaccines and the related studies on the immunomodulatory effects and molecular mechanisms of the body. It provides a reference for the further study of immune adjuvant, immunomodulatory effect and mechanism of TCM polysaccharides, and a new idea for the development of new TCM polysaccharides.

## The Adjuvant Effects on Vaccine of Polysaccharides From TCM

**FIGURE 1:**
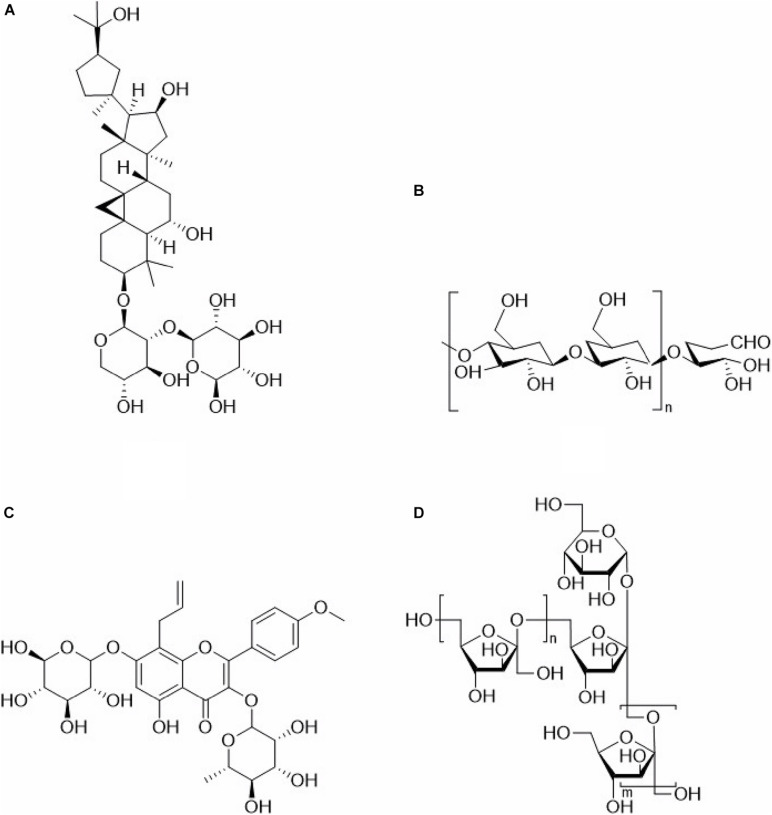
Structural formula of Traditional Chinese medicine polysaccharide. **(A)** Astragalus polysaccharides. **(B)** Ganoderma lucidum polysaccharides. **(C)** Epimedium polysaccharides. **(D)** Achyranthes polysaccharides.

Many TCM polysaccharide can enhance the immune effect of a vaccine, leading to promoted innate immunity and acquired immunity, cellular immunity and humoral immunity. So many TCM polysaccharides can be used as vaccine adjuvants, Table 1 shows adjuvant effects, extraction methods and immunoregulatory activities of some TCM polysaccharides. Because most TCM polysaccharides are crude products, so the chemical structures of only a few TCM polysaccharides were determined, Figure 1 shows the structural formula of some TCM polysaccharides.

**TABLE 1:** Adjuvant effects and immunoregulatory activities of TCM polysaccharides.

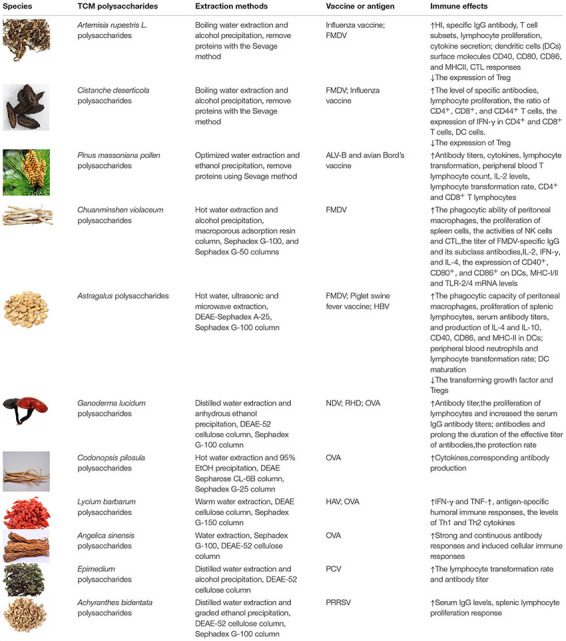

### *Artemisia Rupestris* L. Polysaccharides (ARPS)

Xinjiang *Artemisia rupestris* L. is a traditional medicinal material of ethnic minorities in Xinjiang. It has been used in various therapeutic medicinal materials. The whole plant of Xinjiang AR contains a variety of active ingredients, such as polysaccharides, flavonoids, sesquiterpenes, keto acids, amino acids, and volatile oils. Clinical data shows that it has anti-allergic, anti-bacterial and antiviral effects (Zhang et al., 2017b). Xinjiang ARPS can significantly enhance the level of influenza vaccine HI, specific IgG antibody, T cell subsets, lymphocyte proliferation, and cytokine secretion. At the same time, it can also significantly increase the level of dendritic cells (DCs) surface molecules, down-regulate the expression of Treg cells and enhance CTL responses (Zhang et al., 2017a). Xinjiang ARPS can also significantly increase the level of Foot-and-mouth Disease Vaccines (FMDV) IgG antibody, IgG_1_ and IgG_2a_ subtypes, it has immune synergistic effect on commercial adjuvants of FMDV and long-term protective effect. The study of the mechanism of Xinjiang ARPS on DCs show that it can promote the maturation and function of DCs by stimulating the expression of CD40 and CD86 of DCs, reducing antigen phagocytosis and enhancing the ability to stimulate heterologous T cells (Wang et al., 2019).

### Cistanche Deserticola Polysaccharides (CDPS)

Xinjiang wide desert *Cistanche deserticola* is a commonly used TCM. It belongs to the genus Cistanche in the Lydanaceae, a sand plant, which mainly parasitizes on the roots of desert plants such as Haloxylon ammodendron and Tamarisk. Its main component is polysaccharides, and has many functions such as anti-oxidation, anti-virus and regulation of the immune system (Xiao et al., 2019). Xinjiang wide desert CDPS can significantly enhance the level of specific antibodies of influenza vaccine, promote lymphocyte proliferation and the ratio of CD4^+^, CD8^+^ CD44^+^ T cell.

And significantly induce the expression of IFN-γ in CD4^+^, CD8^+^ T cells and activate DC cells and reduce the expression of Treg (Zhao et al., 2019). FMDV with Xinjiang wide desert CDPS significantly augment the proliferation of lymphocyte, increase the expression of T cell surface molecules, promote the secretion of cytokines IFN-γ and IL-4, and promote the activation of DCs, induce the maturation of DCs by promoting the secretion of DCs surface molecules CD40, CD80, CD86, and MHCII. In short, Xinjiang wide desert CDPS can be used as an effective adjuvant to enhance the humoral and cellular immune response of FMDV (Ba et al., 2017).

### Pinus Massoniana Pollen Polysaccharides (PPPS)

Early studies showed that low-purity Taishan Masson pine pollen polysaccharide (TPPPS) has immunomodulatory effects (Chu et al., 2013). Later studies (Yang et al., 2015) injected TPPPS with subgroup B avian influenza virus (ALV-B) and avian Bord’s, respectively, the results showed that the TPPPS had a gradual improvement in immune function and developmental status. Moreover, we found that chickens vaccinated with TPPPS adjuvanted vaccine showed high level of antibody titers, and the changes in cytokines, lymphocyte transformation, and peripheral blood T lymphocyte count were higher than those in the non-adjuvanted vaccine group (*P* < 0.05). These results indicated that TPPPS has a good immune enhancing effects. The TPPPS and propolis combination group showed the highest antibody titers and IL-2 levels, lymphocyte transformation rate, CD4^+^ and CD8^+^ T lymphocytes (Guo et al., 2014).

### *Chuanminshen violaceum* Polysaccharides (CVPS)

*Chuanminshen violaceum* is a well-tolerated, non-toxic, commonly used Chinese herbal medicine. It is traditionally used as a tonic to strengthen the body and nourish the spleen and lungs (Lin et al., 2019). CVPS enhances specific and non-specific humoral immunity, stimulates T cell proliferation and exhibits anti-mutagenic effects (Lin et al., 2020). The use of CVPS as an FMDV adjuvant can significantly enhance the phagocytic ability of peritoneal macrophages, the proliferation of spleen cells, and the activities of NK cells and CTL, as well as increase the titer of FMDV-specific IgG and its subclass antibodies. In addition, CVPS increased the expression of IL-2, IFN-γ, and IL-4 in CD4^+^ T cells and the expression of IFN-γ in CD8^+^ T cells. Besides, CVPS enhanced the expression of CD40^+^, CD80^+^, and CD86^+^ on DCs. Up-regulates MHC-I/II and TLR-2/4 mRNA levels (Feng et al., 2015).

### *Astragalus* Polysaccharides (APS)

Studies have shown that the *Astragalus* polysaccharide extracted from the root of *Astragalus* has low cytotoxicity or no toxic side effects (Jin et al., 2014). It can enhance the body’s immune function, anti-viral and antibacterial effects (Xie et al., 2016a). Zhang et al. (2010) found that APS can significantly enhance the phagocytic capacity of peritoneal macrophages, proliferation of splenic lymphocytes, serum antibody titers, production of IL-4 and IL-10 in mice immunized with FMDV. The high-dose group significantly enhanced the expression of CD40, CD86 and MHC-II in mouse DCs. APS can significantly increase the antibody level of piglet swine fever vaccine, the percentage of peripheral blood neutrophils and lymphocyte transformation rate (Zhang et al., 2010). It shows that APS has a certain promotion effect on the humoral immune function of piglets. It has also been reported that APS is an effective adjuvant for hepatitis B virus subunit vaccines and DNA vaccines. It can enhance the humoral and cellular immune responses by activating the TLR4 signaling pathway, promoting DC maturation, inhibiting the expression of transforming growth factor and CD4^+^CD25^+^Foxp3^+^ Tregs.

### *Ganoderma lucidum* Polysaccharides *(GLPS)*

*Ganoderma lucidum* is a kind of basidiomycete with various pharmacological activities and has been used in Asian traditional medicine for centuries (Lu et al., 2020). The adjuvant activity of GLPS has been extensively studied *in vitro* and *in vivo* (Cor et al., 2018). The influence of GLPS on the proliferation of peripheral blood lymphocytes of poultry vaccinated with Newcastle disease vaccine (NDV) and the determination of the antibody titer in the serum showed that it significantly promoted the proliferation of lymphocytes and increased the serum IgG antibody titers (Zhang et al., 2014). GLPS significantly stimulates the production of OVA-specific antibodies and triggers OVA-specific T lymphocytes to produce IFN-γ, and induces OVA-specific cytotoxic T cell immunity. This indicats that GLPS, which has no toxic side effects, has a potential immune adjuvant effects (Liu et al., 2016).

### *Codonopsis pilosula* Polysaccharides (CPPS)

*Codonopsis pilosula*, which is sweet and calm in nature, nourishes the spleen and stomach, and promotes blood, is an important tonic TCM. It is commonly used to treat diseases such as uterine bleeding and gastroptosis (Sun et al., 2019). It was reported that CPPS has the following functions including enhancing immune response, inhibiting stress, and other biological activities (Bai et al., 2018). The role of immune regulation is mainly to adjust the body’s immune response by changing immune cells to produce cytokines. Sun conducted a study in 2009, they detected the high level of corresponding antibody production by administering CPPS and OVA with mice (Sun, 2009). In recent years, a large number of reports have shown that CPPS has a strong promoting effect on humoral immunity, and CPPS can promote cellular immunity with low dose.

### *Lycium barbarum* Polysaccharides (LBPS)

*Lycium barbarum* is a famous Chinese medicine and an edible food, which plays a variety of roles in pharmacology and biological processes (Pop et al., 2020). One of its biologically active ingredients is polysaccharides. LBPS is known to have a variety of immunomodulatory functions and immune adjuvant effects (Ding et al., 2019). Synthetic liposomes loaded with OVA and LBPS can induce the expression of IFN-γ and TNF-ɑ and promote antigen-specific humoral immune responses, leading to high levels of Th1 and Th2 cytokines (Su et al., 2014). Hepatitis A antigen (HAV) was mixed with LBPS and injected intraperitoneally into the mice. The results show that LBPS as an adjuvant for hepatitis A vaccine has the best immune effect, and it also has a synergistic effect with aluminum adjuvant.

### Other Polysaccharides Derived From TCM

*Angelica sinensis* polysaccharide is used as a H9N2 vaccine adjuvant to improve blood coagulation (Gu et al., 2020). *Epimedium* polysaccharide has a significant adjuvant effect on the inactivated porcine circovirus vaccine (PCV) in mice, which can significantly increase the lymphocyte transformation rate and antibody titer of immunized mice (Fan et al., 2015). *Achyranthes bidentata* polysaccharides can significantly increase serum IgG levels, splenic lymphocyte proliferation response, and significantly increase the titer of porcine reproductive and respiratory syndrome virus (PRRSV) vaccine antibodies (Liu et al., 2013).

## Immunoregulatory Mechanism of Polysaccharides From TCM

**FIGURE 2:**
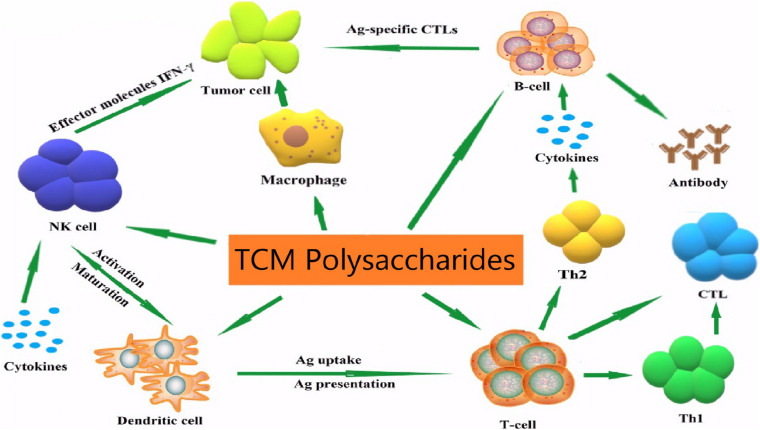
Immune regulatory mechanisms of TCM polysaccharides.

Studies have shown that many TCM polysaccharides have immunomodulatory effects (Kiddane and Kim, 2020; Figure 2) and they are natural immunomodulators which can activate immune cells such as T/B lymphocytes, macrophages and natural killer (NK) cells, promoting the release of a variety of cytokines and the production of antibodies (Borazjani et al., 2018) and activating the complement system (Bao et al., 2015). However, the regulation of immune cells by TCM polysaccharides is closely related to the regulation of cell signal transduction functions. Through mediating cell signal pathways and regulating intracellular gene expression, the regulation of apparent traits is achieved (Abdala Diaz et al., 2019). The following content summarizes the relevant research on the immunomodulatory effect of TCM polysaccharides on animal organisms and the molecular mechanism in recent years, and provides a reference for more in-depth research on the immunomodulatory effect and mechanism of TCM polysaccharides, and also provides new ideas for the development of new TCM polysaccharides.

**FIGURE 3:**
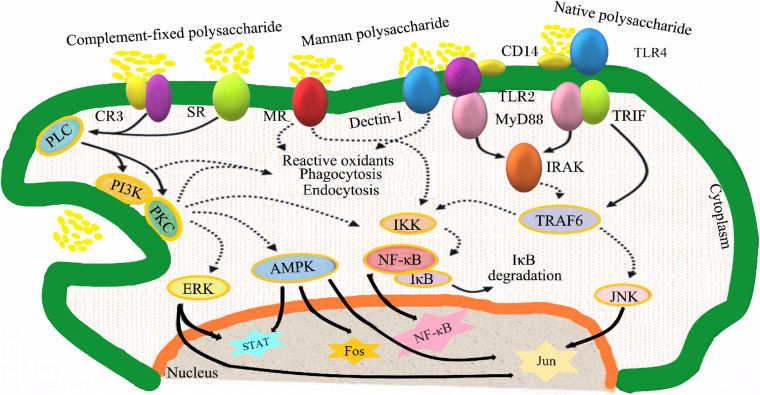
Signaling pathways involved in macrophage activation by polysaccharides. CR3, complement receptor 3; SR, scavenger receptors; MR, mannose receptors; Dectin- 1, dendritic cell-associated C-type lectin-1; CD14, cluster of differentiation antigen 14; TLR2, Toll-like receptor 2; TLR4, Toll-like receptor 4; PLC, phospholipases C; TRIF, Toll/IL-1 domain containing adaptor inducing interferon β; MyD88, myeloid differentiation factor 88; PI3K, phosphatidylinositol 3 kinase; PKC, protein kinase C; IRAK, interleukin-1 receptor associated kinase; IKK, inhibitor of nuclear factor kappa-B kinase; TRAF6, tumor necrosis factor receptor-associated factor 6; NF-κB, nuclear factor kappa-B; IκB, inhibitor of nuclear factor kappa-B; MAPK, mitogen-activated protein kinase; ERK, extracellular signal-regulated kinase; STAT, signal trans- ducers and activators of transcription; JNK, c-Jun N-terminal kinase.

Many studies have found that TCM polysaccharides regulate the function and metabolism of immune cells through multiple signal transduction pathways. This is an important mechanism for TCM polysaccharides to exert immunomodulatory effects, including the signal of polysaccharides to activate macrophages (Figure 3) and T/B lymphocytes signal pathways, regulate natural killer cells signal pathways, and regulatory mechanisms for activation of the complement system.

### Signal Pathways Regulating Macrophages

Macrophages express a variety of pattern recognition receptors on the surface. These pattern recognition receptors can recognize and bind to plant polysaccharides, and transmit signals into the cell through various signal transduction pathways, causing a series of signal cascades in the cell to regulate related gene expression.

#### TLR2/4 Mediated Signal Transduction Pathway

Toll-like receptors (TLRs) are a class of transmembrane protein-like pattern recognition receptors, which are widely expressed on the surface of dendritic cells, macrophages, neutrophils and lymphocytes (Roeder et al., 2004). Among the members of the Toll-like receptor family discovered so far, only TLR2 and TLR4 can bind glycosylated ligands, and both play important roles in the innate and adaptive immune systems. After the plant polysaccharide ligand binds to TLR2/4, it activates the tumor necrosis factor receptor related molecule-6 (TRAF6) through the MyD88-mediated signaling pathway or the Toll-like receptor-related interferon activating factor (TRIF) signaling pathway (Uchiyama et al., 2015). And then, the activated TRAF6 can activate the signal transduction of MAPK and NF-κB pathways, respectively (Yambe et al., 2020).

MAPK is a highly conserved serine/threonine protein kinase that can be activated by extracellular stimulation (Yuan et al., 2021). The MAPK family members in mammalian cells mainly include ERK, JNK, and p38. When the cell is stimulated, MAPKKK is activated by phosphorylation, which in turn continues to activate MAPKK, and then activates MAPK (including ERK, p38, JNK) through double-site phosphorylation, and the phosphorylated transcription factors produced by the activated MAPK enter the nucleus to Regulate the transcription of related genes (Yeom et al., 2015). Studies have shown that *Poria cocos* polysaccharides can significantly promote the expression of iNOS gene and the secretion of NO in mouse RAW264.7 cells, and the p38 signaling pathway mediated by TLR4 is a signaling pathway for *Poria cocos* polysaccharides to activate macrophages (Lee et al., 2004).

NF-κB transcription factor is a kind of structurally related transcription factor in eukaryotes, which regulates the expression of more than 150 genes in cellular processes (Thu and Richmond, 2010). When the cell is stimulated, the IκB kinase (IKK) complex is activated, and IκB is phosphorylated and dissociated from NF-κB under the catalysis of IKK, thereby converting NF-κB into an activated form, and the activated NF-κB is transported (Maijer et al., 2015). In the nucleus, it combined with related DNA sites to promote the transcription of target genes, thereby affecting the transcription of a variety of inflammatory mediators and cytokines. Research by Li and Xu (2011) showed that *Polyporus umbellatus* polysaccharides can activate mouse macrophages through the NF-κB signaling pathway mediated by the TLR4 receptor.

#### The Signal Pathway Mediated by CD14 and CR3

Leukocyte differentiation antigen 14 (cluster of differentiation antigen 14, CD14) is a glycoprotein that exists on the surface of monocytes or in the plasma. It is a high affinity lipopolysaccharide (LPS) receptor in the body (Wright et al., 1990). CR3 is a heterodimeric glycoprotein composed of two subunits of CD11b and CD18 combined with non-covalent bonds. It is a member of the leukocyte β2 integrin family and can recognize β-glucan (Ross and Vetvicka, 1993). CD14 and CR3 activate phospholipase (PLC), then activate protein kinase (PKC) and phosphatidylinositol-3-kinase (PI3-K), and regulate the expression of related genes through MAPK or NF-κB signaling pathways (Schepetkin and Quinn, 2006). *Platycodon grandiflorum* root polysaccharide can activate iNOS transcription and NO production in macrophages, and activate macrophages through CD14 and CR3 (Shan et al., 2020).

#### The Signal Pathway Mediated by Mannose Receptors (MR)

MR is a member of the C-type lectin-like receptor family, which is mainly expressed by macrophages and can recognize mannose, L-fucose and L-Acetyl Glucosamine (Fang et al., 2021). Guo et al. found that *Rheum palmatum* polysaccharides containing N-acetyl glucosamine residues stimulate macrophages to secrete TNF-α through MR receptors.

#### The Signaling Pathway Mediated by Scavenger Receptors (SR)

SR is a transmembrane glycoprotein with diverse structures that mainly exists on the surface of macrophagesand dendritic cells, it can recognize and bind Gram-negative bacteria lipopolysaccharide etc. (Murphy et al., 2005). Studies have shown that the pathway for SR receptors to activate macrophages may be consistent with CR3 receptors (Ilchmann et al., 2010). Fucoidan can significantly promote the release of NO in RAW264.7 cells of wild-type mice (containing SR gene), and have no activating effect on the macrophages of SR gene-deficient mice, indicating that fucoidan activate macrophages to release NO through SR receptors (Nakamura et al., 2006).

#### Dectin-1 Mediated Signaling Pathway

Dectin-1 is a type II transmembrane receptor composed of 4 subunits (Brown et al., 2002). The extracellular area is a C-type lectin-like region, and the cytoplasm is a tyrosine active area (Bono et al., 2020). In monocytes/macrophages, β-glucan can be recognized. After Dectin-1 binds to the ligand, the tyrosine activation motif (ITMA) in the cytoplasm undergoes tyrosine phosphorylation under the action of tyrosine (Src) family kinases, and activates tyrosine kinase (Syk), then activates intracellular signaling pathways to cause cellular responses (Guasconi et al., 2018). *Ganoderma lucidum* spore polysaccharides can activate macrophages to promote the release of TNF-α through Dectin-1 (Guo et al., 2009).

### Molecular Channels Regulated by T/B Lymphocytes

The molecular channels of plant polysaccharides to activate T lymphocytes are mainly through the T-cell receptor (TCR)/CD3 complex receptor-mediated signaling pathway, and the downstream MAPK signaling (Dong et al., 2019). The regulatory pathway for plant polysaccharides to activate B lymphocytes is mainly combined with the IgM/CD79 complex receptor or TLR2/4 on the cell surface, and the downstream of the pathway is mainly regulated by the two signal transduction pathways of MAPK and NF-κB (Wu et al., 2019). *Astragalus* polysaccharides (APS) can stimulate the proliferation of B lymphocytes in BALB/c mice (Shao et al., 2004).

### Signal Pathways Regulating Natural Killer Cells

Natural killer cells can be activated through a variety of ways, including CD3 molecules on the membrane surface and a variety of cytokines (Sheng et al., 2018). Natural killer cells have IL-2 affinity receptors on the surface, so IL-2 can enhance the activity of natural killer cells. Soluble β-glucan can significantly enhance the killing effect of natural killer cells on K562 cells, and this enhancement is inhibited by CR3 antibody, indicating that the binding recognition site of β-glucan and natural killer cells may be CR3 (Vetvicka et al., 1997).

### The Regulatory Mechanism for Activation of the Complement System

The complement system can be activated by three pathways: the classic pathway, the alternative (bypass) pathway, and the lectin pathway. The classical pathway is the first antibody-mediated activation pathway discovered. The process is that IgG and IgM molecules bound to the antigen are used as activators to bind to complement (C)1q, and sequentially activate C1r, C1s, C2, C4, and C3. The formation of a cascade enzymatic reaction between C3 convertase and C5 convertase; the alternative pathway does not rely on antibodies, and C3 is directly activated by microorganisms or foreign substances (Liu et al., 2011).

## Conclusion and Perspectives

TCM polysaccharides have been a research hotspot in recent years. Studies have shown that polysaccharides have a variety of biological effects. Moreover, as a natural active ingredient, TCM polysaccharide has the characteristics of non-toxic, harmless, non-residue, and non-drug resistance. It has a good adjuvant effect for many human and animal vaccines, and possesses a wide range of vaccine development prospect.

Numerous studies have shown that TCM polysaccharides regulate the function and metabolism of immune cells through multiple signal transduction pathways. Regulatory mechanism including polysaccharides to activate macrophages, the signal pathway of T/B lymphocytes, regulate the signal pathway of natural killer cells, and activate the complement system. This mechanism is very important for TCM polysaccharides to exert immunomodulatory effects.

However, due to the limitations of extraction technology for current polysaccharides, TCM polysaccharides are mostly crude products with low purity. This has led to the production of various TCM polysaccharides reported by many researchers. The recommended dosage is different, and it also brings great difficulties to the in-depth study of the molecular mechanism of TCM polysaccharides regulating animal immunity. At the same time, China is rich in plant resources, TCM polysaccharides are diverse, and there are considerable differences in the structure of polysaccharides from different sources. Further research and exploration are needed for their functions and mechanisms.

## Author Contributions

DW and YL wrote the manuscript. All authors contributed to the article and approved the submitted version.

## Conflict of Interest

The authors declare that the research was conducted in the absence of any commercial or financial relationships that could be construed as a potential conflict of interest.
